# Medical Images Fusion with Patch Based Structure Tensor

**DOI:** 10.2174/1874120701509010199

**Published:** 2015-08-31

**Authors:** Fen Luo, Jiangfeng Sun, Shouming Hou

**Affiliations:** School of Computer Science and Technology, Henan Polytechnic University, Jiaozuo, 454000, China

**Keywords:** Medical image fusion, patch similarity, structure tensor, wavelet decomposition

## Abstract

Nowadays medical imaging has played an important role in clinical use, which provide important clues for medical diagnosis. In medical image fusion, the extraction of some fine details and description is critical. To solve this problem, a modified structure tensor by considering similarity between two patches is proposed. The patch based filter can suppress noise and add the robustness of the eigen-values of the structure tensor by allowing the use of more information of far away pixels. After defining the new structure tensor, we apply it into medical image fusion with a multi-resolution wavelet theory. The features are extracted and described by the eigen-values of two multi-modality source data. To test the performance of the proposed scheme, the CT and MR images are used as input source images for medical image fusion. The experimental results show that the proposed method can produce better results compared to some related approaches.

## 1. INTRODUCTION

In the resent years, medical imaging plays an important role in various applications in clinical use, such as pathology analysis, clinical diagnosis or healing examinations etc. In view of the characteristic of medical image, we must get good quality image and complete relative medical image to ensure the diagnostic correctness. While one type modality input source image provides a certain kind of information about the human body and some other information are provided by some other kinds of the source images. For example, dense structures information like the bones and implants with less distortion are provide by computed tomography (CT) and X-ray while physiological changes detection information requires MRI imaging, which can visualize normal and pathological soft tissue. Furthermore, the information of blood flow is provided by PET scans, which is efficient with low spatial resolution case. Therefore, image fusion technology cast a light to integrate and present the different kinds of information from two or more imaging modality into a single image, which is more appreciated by hospital. Hence, the fusion of the medical images is becoming necessary nowadays which is more suitable for human perception and diagnoses by doctors.

Numerous techniques have been proposed in past decades to deal with medical image fusion. The most classical way is to select or average the density pixel-by-pixel from the input medical source images [1-4]. The statistical or decomposition methods are also empolyed. Some typical scheme includes medical image fusion methods based on wavelet analysis [5], weighted averaging to complex multi-resolution pyramid [6] or neural network approach [7, 8]. But for image fusion, wavelet decomposition is still a popular and important one.

In this paper, a novel algorithm for medical image fusion is proposed which employ a tensor matrix to describe some important feature information. We define a patch similarity based tensor matrix. Then it is used to extract local features from low frequency and high frequency wavelet coefficients. On the basis of these features, an average fusion rule is established. The remaining part of this paper is organized as follows: we first introduce wavelet framework and the classical structure tensor, and then we modify the new structure tensor by using the nonlocal mean filter in Section 2. In Section 3, we present a patch based fusion approach within the multi-resolution wavelet theory. Section 4 shows some experimental results of medical image fusion with comparison with some relative methods. A brief conclusion is given in Section 5.

## 2. WAVELET FRAMEWORK AND PATCH BASED STRUCTURE TENSOR 

## 2.1 Multi-resolution Wavelet Framework

Wavelet analysis has many advantages and is an important tool for various image processing tasks, including medical image fusion. One essential step in wavelet image fusion is the combination of wavelet coefficient. Namely, the final results mainly depends on the merge of the two families coefficients derived the input source images. Fig. (**1**) illustrates a flowchart through which medical images are fused. The difference between different approaches lies in two aspects. The first aspect is the way to extract the features. And the second aspect is the fusion rule.

For two source medical images, we denote them as A and B respectively. Also the goal image of image fusion is denoted as image F. The main steps of the proposed algorithm are as follows:

1. Image A and B are performed k-level discrete wavelet transform are applied two source input images A and B. After the wavelet decomposition, the high frequency and low frequency coefficients are obtained. A single sub-band is yielded for a certain level. The low-frequency sub-bands of image A is denoted $W_{L}^{A}(p)andW_{L}^{B}(P)$for image B. There are three high frequency sub-bands for a certain level k, LH sub-band, HL sub-band and HH sub-band. We denote them as a general formation as $W_{ijk}^{A}(p)andW_{ijk}^{B}(p)$, where j=LH,HL,HH.
2. After the wavelet decomposition of two input source medical images, the low-frequency coefficients and the high-frequency coefficients are fused with a fusion rule. The feature information, which are mainly composed in high frequency coefficients, are used for fusion is very important. The corresponding fusion rule also is critical for fusion result. In the next sub-section, we will give more details. After the combination of wavelet coefficients, the low-frequency sub-bands is denoted by$W_{L}^{F}(p)$ and the high frequency sub-bands are $W_{ijk}^{F}(p)$, j=LH,HL,HH.
3. As an inverse processing of wavelet decomposition, the low-frequency $W_{L}^{F}\left(p\right)$and the high-frequency coefficients are used to reconstruct the fused image F by a standard discrete wavelet transform.

In the next sub-section, the way to extract feature and the fusion rule will be given.

## 2.2 Structure Tensor

Gradient is a very important mathematics tool for image processing. It can be used for edge detection, image segmentation and some other tasks. However, it is not robust for noise. To deal with this problem, averaging is a norm way to suppress noise. Unfortunately, the sign of gradient may be opposite, which means it can make cancellation effect [9]. To solve this problem, we introduce an alternative tool, structure tensor. For a discrete imageI(x,y), its two first order derivatives with respect two directions is used to construct the gradient vector $\nabla I={\left(I_{x},I_{y}\right)}^{T}$, where T is the transpose. And we define the outer product of gradient vector and its transpose as the (initial) structure tensor:

(1) $$J_{0}=\nabla I\nabla I^{T}=\left(\begin{array}{cc} I_{x}^{2} & I_{x}I_{y}\\ I_{x}I_{y} & I_{y}^{2} \end{array}\right)$$

It is easy to justify that $J_{0}$is positive semi-definite matrix. After a standard computation, we can know its two eigen-values are $\lambda_{1}={\left|\nabla u\right|}^{2}and\lambda_{2}=0$respectively. The eigen-vector corresponding to $\lambda_{1}$shares the same direction with∇uwhile the other eigen-vector $\nu_{2}$ is orthogonal to∇u. A normal way to suppress noise is to apply the convolution of the components of $J_{0}$with a Gaussian kernel$K_{\rho }$ (Gaussian smoothing), by which $J_{0}$ is extended to the linear structure tensor$J_{\rho }=J_{0}*K_{\rho }$. 

## 2.3 Nonlocal Structure Tensor

It is come to a common that classical Gaussian filter blurs and dislocates structures. The same situation happens for linear structure tensor. The main reason for this undesired effect is the weight of Gaussian filter only depends on the distance between two pixels. The weight is fixed for the fixed distance and it cannot adapt to the structure of the input data. When the filtering is applied, some important features may be smoothing away. Some improvements have been made by using some adaptive structure tensor, such as the nonlinear structure tensor in [10, 11], bilateral-based structure tensor in [12]. 

However, the above mentioned technologies only use the local structure information and neglect the relations between the pixels far away. The relation may be important especially when the two pixels have similar structure, even though the distance between them is far. In this case, a larger weight should be given. Therefore, we use the nonlocal means (NLM for short) filter, to construct a patch based structure tensor. The key idea of NLM is that the noised contained in an image may be smoothed away by averaging them as images contain repeated structures [13]. After computing the patch similarity, which is define as the weight sum of the difference of two patches with the same size, weight is appointed. If the structure of the neighbor is similar, the larger weight is given. 

A standard NLM can be calculated as below. For a discrete noisy imagev=v(X)|X∈I, the filtered value

NLM(v(X)), is the weighted average of all the pixel in the image,

(2) $$NLM(v(X))=\sum_{Y\in I}\omega (X,Y)v(Y).$$

The weights $\omega {\left(X,Y\right)}_{Y}$ satisfy the usual conditions $0\leq \omega {\left(X,Y\right)}_{Y}\leq 1and\sum_{Y\in I}\omega (X,Y)=1$. 

The weights ω(X,Y) describe the similarity of the two pixels X and Y. The Neighborhood of a pixel is usually defined as a square window whose radius is r. These weights are calculated as

(3) $$\omega (X,Y)=\frac{1}{Z(Y)}e^{-d(X,Y)}$$

where

(4) $$Z(I)=\sum_{J\in I}e^{-\frac{d(X,Y)}{h^{2}}}$$

where h is a parameter to control filter degree. d is a scalar defined as :

(5) $$d(X,Y)=G_{\rho }{\left\|v(N_{X})-v(N_{Y})\right\|}_{r}^{2}$$

where G_ρ_ is a normalized Gaussian weighting function with zero mean and ρ standard deviation. There are two aspects that patch based filtering is prior to some classical filters such as Gaussian filter and bilateral filter. One aspect is NLM consider the information between two far away pixels. The long distance relation is exploited. The second one is that the local structure, which is contained in a local window region, is used to extract geometrical feature. In a word, the weight of NLM mainly depends on the similarity of the two patches, instead of that of two isolated pixels. Therefore, it obtain a better details-preserving denoising results. To improve the robustness of the original structure tensor, a patch based structure tensor($J_{NLM}$) defined as below:

(6) $$J_{NLM}=NLM(J_{0})=\left(\begin{array}{cc} J_{11} & J_{12}\\ J_{12} & J_{22} \end{array}\right)$$

Two eigne-vectors of matrix $J_{NLM}arev_{1}andv_{2}$. And two vectors are orthonormal. At the same time, they are paralled to

(7) $$\left(J_{11}+J_{22}-\frac{2J_{11}}{\sqrt{({\left(J_{11}-J_{22}\right)}^{2}+4J_{12}^{2}}}\right).$$

Two eigen-values are given by

(8) $$\mu_{1}=\frac{1}{2}\left[J_{11}+J_{22}+\sqrt{({\left(J_{11}-J_{22}\right)}^{2})+4J_{12}^{2}}\right]$$

and

(9) $$\mu_{2}=\frac{1}{2}\left[J_{11}+J_{22}-\sqrt{({\left(J_{11}-J_{22}\right)}^{2})+4J_{12}^{2}}\right]$$

Eigen-direction and eigen-value contain the important information of the local features. For example, The highest grey value fluctuations orientation is indicated by $v_{1}$, while the coherence direction is given by $v_{2}.\mu_{1}≅\mu_{2}$implies the variation in a local region is very small and we can assert that it is a homogenous region.$\mu_{1}\gg \mu_{2}=0$s implies the variation in one the main direction is strong and the variation in another eigen-vector direction is weak. A straight edges or flow-liked region shows this situation.$\mu_{1}\geq \mu_{2}\gg 0$implies that a corner may be detect. We use the definition of local coherence measure as

(10) $$\phi ={\left(\mu_{1}-\mu_{2}\right)}^{2}$$

The idea of new structure tensor can be extended to some relative image processing tasks, as illustrated in [14]. 

## 3. MEDICAL IMAGE FUSION WITH NONLOCAL STUCTURE TENSOR

In a standard multi-resolution wavelet theory, the low-frequency sub-band LL represents the approximation part while the detail information is contained in three high-frequency sub-bands. Therefore, we devise two different strategies for these two types of coefficients fusion. Once the fused coefficients are obtained, the fused medical image can be obtained after an inverse wavelet transform. 

## 3.1 Low-frequency Sub-band Fusion Rule

In our experiment, we apply widely used average method for low-frequency sub-band coefficients:

(11) $$W_{L}^{F}(p)=k_{1}*W_{L}^{A}(p)+k_{2}*W_{L}^{B}(p)$$

Where parameters k_1_ and k_2_ are fixed as k_1_=0.75 and k_2_=0.25.

## 3.2 The Fusion of High-frequency Sub-band

An essential step in medical image fusion is the way to combine high-frequency sub-bands. The features, such as edges and lines, produce larger coefficients. An ordinary way for fusion rule is the adaptive weighted average (WA) scheme, in which the fused high-frequency coefficients are the weighted sum of that of the source images. An alternative way is choose-max (CM) scheme, which uses directly the coefficient with the larger absolute value. In out setting, a patch based nonlocal structure tensor is used to measure local geometrical information. The eigen-values of patch based structure tensor show the local shape information, which are critical clues for fusion. Based on the discussion above, a novel medical image fusion rule is given as follows:

(12) W(p)ijkF=ωijk∗WijkF(p)+(1−ωijk)∗WijkF(p)

where $\omega_{ijk}$is the weighted coefficients defined by

(13) $$W_{ijk}=\frac{\phi_{ijk}^{A}}{\phi_{ijk}^{A}+\phi_{ijk}^{B}}$$

In equation (13), $\phi_{ijk}^{A}$is the local coherence measure for a certain high sub-band in k-level decomposition of image A. The definition of $\phi_{ijk}^{B}$ is similar with that of image B.

## 4. EXPERIMENTAL RESULTS

To test the performance of the proposed scheme, we tested our method with CT and MR images. To do the quantitative analysis of experimental results comparison. Energy of image gradient (EOG) is used as quantitative comparison. We compare our method with two schemes: weighted average (WA) and choosing gradient max (CGM) fusion scheme.

The whole fused image is shown in Fig. (**2**). To further investigate the detail performance, we also give two zooming parts. In our experiments, the wavelet decomposition is applied to three methods with three levels. The wavelet basis function used is ’db3’.

**Fig. (2):**
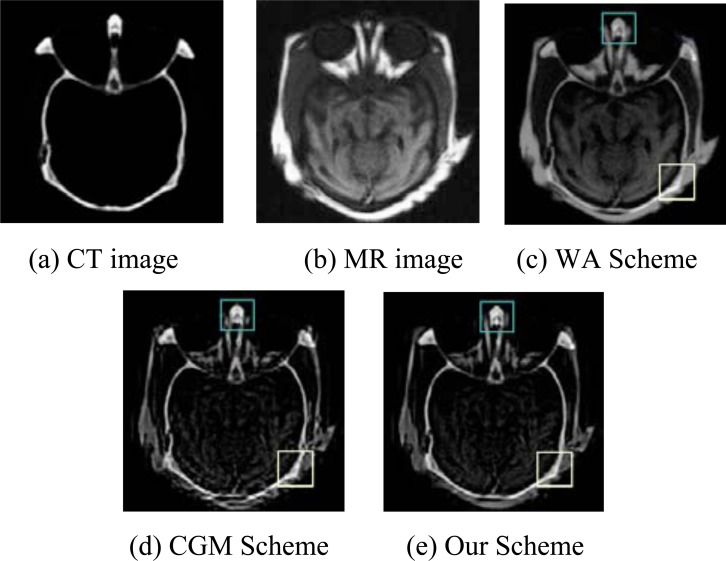
Fusion of medical images.

Fig. (**2**) illustrated the fusion results of CT and MR medical images of the same brain area. The fusion results by different schemes are shown in Fig. **2**(**c**)- Fig. **2**(**e**). The search window is 11 × 11 and the similar window size is 5 × 5. AW scheme produces a fuzzy effect when compared with the other methods. As displayed in Fig. (**3**), patch based method produces a more smoothing effect for homogenous region. In Fig. (**4**), it is easy observed that AW scheme produces a lower contrast. Ghost occurs near edges in the fused image with CGM scheme. Our scheme preserves edges and keeps relative smooth. EOG data is reported in Table **1**.

## CONCLUSION

In this paper, a patch based structure tensor is defined. Then it used as a tool to extract information. The fusion rule uses adaptive weighted function of eigen-values. The proposed performances better when compared some related methods.

## Figures and Tables

**Fig. (1):**
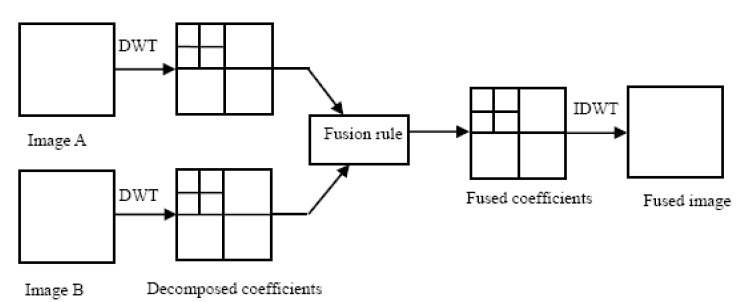
Wavelet-based image fusion framework.

**Fig. (3):**
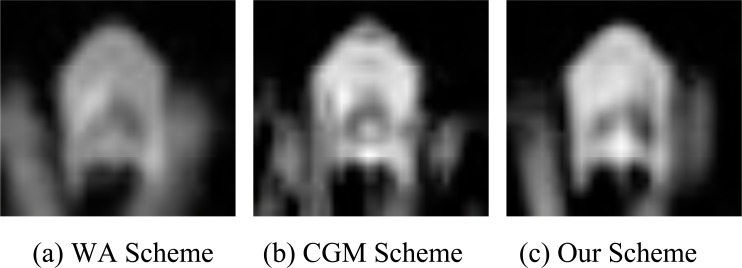
Center part of the fusion results.

**Fig. (4):**
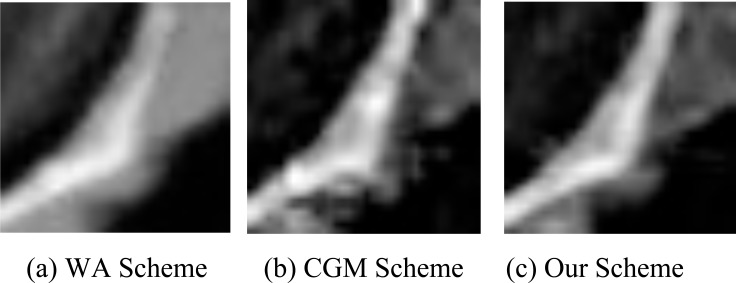
Right part of the fusion results.

**Table 1.** Comparison of the EOG data.

	WA	CGM	Our Scheme
CT/MR	4.0363	4.6583	5.1172
